# Supplementary figures and images for: Genome-Wide Identification and Expression Analysis of the Metacaspase Gene Family in Gossypium Species
Source: Genes (Basel). 2019 Jul 12;10(7):527. doi: 10.3390/genes10070527 (PMC6679041; doi:10.3390/genes10070527)

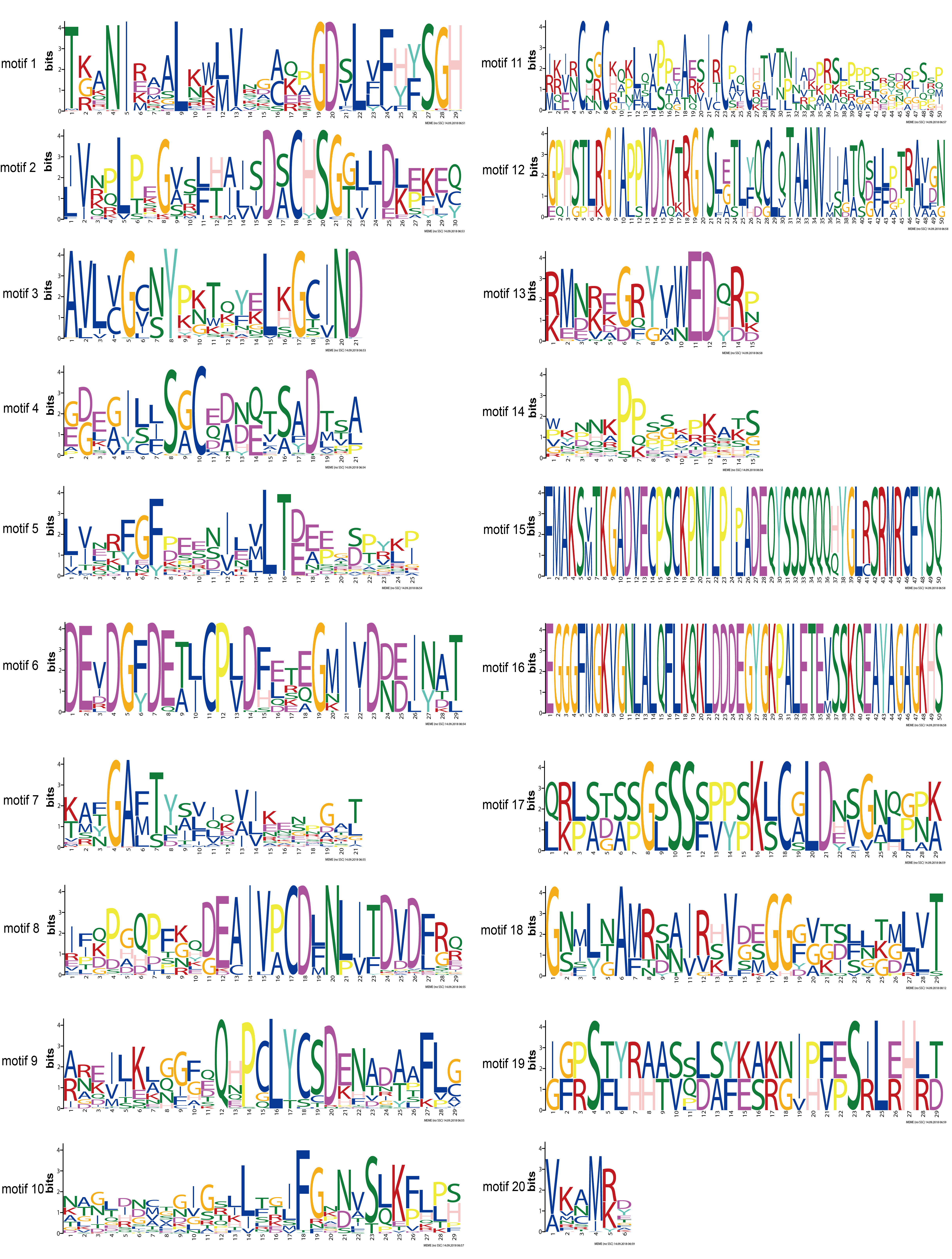

Supplement: Supplementary file 1 [file genes-10-00527-s001.zip › supplementary files/Figure S1.tif]
